# Antrum Tubæ of Rœderer

**Published:** 1845-09

**Authors:** 

**Affiliations:** Glasgow


*Antrum Tubæ of Rœderer.* By Dr. Ritchie, Glasgow.—This modification of the structure of the curved and sacculated distal extremity of the Fallopian tube, which was first described by Rœderer, and afterwards by Montgomery, has been suggested by Dr. Ritchie to be pathological. He says, "In occasional instances, both of women who have not borne children, and of such as have been mothers, one, and sometimes two portions of this vesical-like process project from the line of the tube, in the form of well-defined chambers or recesses. These affect a globular shape externally, and are sometimes so thin as to be translucent, while, internally, the muscular fibrils of the tube are gathered into bundles around the neck or orifice of the little chamber,—the whole forming a structure not unlike a miniature hernial sac, and communicating the impression, that the muscular layer of the canal had gradually given way under some mechanical force, such as that of the tube frequently and strongly compressed,—distending the mucous lining, and separating the muscular fibres, till at length the inner and outer, or peritoneal, coats were protruded beyond the muscular layer, after the manner of a direct ventral hernia, the fleshy fibrils of the latter being found arranged around its neck.—*Lond. Med. Gaz.*

